# The perspectives of homeless people using the services of a mobile health clinic in relation to their health needs: a qualitative study on community-based outreach nursing

**DOI:** 10.1177/17449871231159595

**Published:** 2023-04-27

**Authors:** Etienne Paradis-Gagné, Marie-Claude Jacques, Pierre Pariseau-Legault, Houssem Eddine Ben Ahmed, Ioana Ruxandra Stroe

**Affiliations:** Assistant Professor, Faculty of Nursing, Université de Montréal, Montréal, Canada; Associate Professor, Faculty of Medicine, School of Nursing, Université de Sherbrooke, Sherbrooke, Canada; Associate Professor, Department of Nursing, Université du Québec en Outaouais, Quebec, Canada; Postdoctoral Fellow, School of Sociological and Anthropological Studies, University of Ottawa, Ottawa, Canada; Registered Nurse, Faculty of Nursing, Université de Montréal, Montréal, Canada

**Keywords:** community health nursing, critical ethnography, homelessness, mobile clinic, nursing outreach

## Abstract

**Background:**

Significant social and health issues are associated with homelessness. Negative experiences with the healthcare system are also frequent and cause people experiencing homelessness to avoid health services.

**Aims:**

The purpose of this study was to (1) explore participants’ health needs concerning outreach nursing services and (2) describe the perceptions and preferences of people who access this form of community-based intervention.

**Methods:**

We conducted a critical ethnography with semi-structured interviews of 12 people experiencing homelessness who receive the services of a nurse-led mobile clinic, and 60 hours of observation during the provision of these services.

**Results:**

Our results describe the perspectives of people experiencing homelessness in three main categories: (1) worrisome health and social needs, (2) non-use of healthcare and (3) what connects us to health services.

**Conclusions:**

Timely access to healthcare is an important issue for people experiencing homelessness. Nurse-led clinics meet needs that go far beyond health issues.

## Introduction

Homelessness and social inequality are significant issues in Canada, as in other countries across the world. For instance, there are an estimated 2.5–3.5 million homeless people in the United States annually and 4 million for the European Union (Fazel et al., 2014). In a 2018 census of homeless people in 61 Canadian communities, 32,005 people reported being homeless. Of these, 25,216 had no fixed address every night (absolute homelessness) and 6,789 were in a transition programme (Employment and Social Development Canada, 2019). In Canada, we also know that homelessness is increasingly affecting a wide variety of sub-populations: teens, LGBTQ2S-identified individuals, people who are immigrants, Indigenous, families and veterans (Gaetz et al., 2016). All these groups have distinct characteristics and specific health needs. In the greater Montréal area (province of Québec, Canada), where homelessness has been a growing phenomenon in recent decades, a 2019 census indicated that more than 3000 people were homeless (Gouvernement du Québec, 2019).

According to the literature, homelessness is associated with different factors such as substance misuse, living in traumatic relationships, poverty and mental health problems (Barrett et al., 2011; Bhui et al., 2006; O’Carroll and Wainwright, 2019). This phenomenon, whose causes are multifactorial (socio-economic, political, legal and societal), increases both social and health inequities, in addition to complicating access to primary healthcare (Gaetz et al., 2016; Luchenski et al., 2018). This is a shocking and confronting reality for Canada, a country where the healthcare system is allegedly required to meet the criteria of public administration, comprehensiveness, universality and accessibility for every citizen (Canada Health Act, 1985).

Major psychosocial and health issues are associated with homelessness; indeed, many homeless people reported having physical and mental health problems and needs that affected their quality of life (Barrett et al., 2011; Bhui et al., 2006). However, studies have shown that these populations have negative experiences within a healthcare system that marginalises, stigmatises and discriminates against them (Hudson et al., 2010; Omerov et al., 2020; Purkey and MacKenzie, 2019). In this respect, many authors stressed the importance of building appropriate relationships between homeless people and healthcare providers that are based on trust, compassion, respect and fairness (Darbyshire et al., 2006; Purkey and MacKenzie, 2019; Yu et al., 2019).

In response to the health needs of people experiencing homelessness, different interventions have been developed, notably nurse-led clinics (Roche et al., 2017; Savage et al., 2008) and mobile health clinics (Malone et al., 2020). This type of outreach service can facilitate homeless people’s access to healthcare by providing it directly in their living environment. Yet, until now, these people have had very few opportunities to be heard about their perspectives concerning the ability of these services to match their needs (Kiser and Hulton, 2018; Randall et al., 2017).

In this regard, the aim of our research was to study the points of view of people experiencing homelessness who use the nursing services of a mobile health clinic. The objectives of the project were to (1) explore participants’ health needs for outreach services and (2) describe the perceptions and preferences of people who access this form of community-based intervention.

## Methodology

### Research design

For this project, we selected critical ethnography as our research methodology (Madison, 2019). The purpose of this qualitative approach is to shed light on the social inequalities existing in society and in healthcare settings, as well as the effects and consequences of the structural discrimination imposed on marginalised populations. According to Madison (2019) and Luchenski et al. (2018), we can think specifically of people experiencing homelessness, incarcerated people, drug users and sex workers, who may be structurally affected by mechanisms of social exclusion, racism, colonialism, transphobia and sexism, for example.

We opted for this methodological approach to emphasise the social and political dimensions of the problem of homelessness and the difficulties of access to health services for people experiencing homelessness. In terms of political dimensions, one can think of decreased funding in social programmes for access to employment and housing, budgetary restraint measures (neoliberal policies and austerity) and cuts in healthcare, among others (Donnan, 2014; Fazel et al., 2014; Gaetz et al., 2016; Stonehouse et al., 2022).

### Research setting

This research was conducted in partnership with a non-profit organisation in the community in Montréal, Canada. Data was collected through the organisation’s mobile clinic, which goes to different neighbourhoods to provide care to people experiencing homelessness. This clinic consists of a team of nurses, street workers and volunteers who offer services in a truck with a consultation room and medical equipment. The services offered are diverse and may vary depending on the clients’ needs (primary care, wound care, sexually transmitted disease screening and blood tests, distribution of safe injection equipment and condoms, referrals to medical clinics, etc.).

### Sampling

To recruit participants, we sent an email with information about the project to the leaders of the mobile clinic’s community partner organisations (shelters and day centre). Participants were compensated in cash for their contribution to the interview. A recruitment poster was also posted inside the clinic. Recruitment of participants was therefore facilitated with the help of community workers (key-informants), who disseminated the information and invited people to come and meet us. We also used the snowball method (Morgan, 2008): people who participated in the project recommended to their friends and family that they come for an interview. Word of mouth and the regular presence of the research team in the field thus contributed largely to the recruitment of participants.

### Data collection

Semi-structured interviews were conducted with individuals who are currently (or have been) experiencing homelessness and who use the mobile clinic’s services. The interviews were held in shelters, community organisations or parks, depending on the participant’s preference. Interviews were conducted in English and French, and an interview guide was used (see Table 1). The French interviews were translated into English, and then compared to the original version to ensure authenticity. We recruited a sample of 12 participants (see Table 2). Interviews were digitally recorded before being transcribed. A field journal (Madison, 2019) was kept throughout the research to document the researchers’ reflections.

**Table 1.** Interview questions.

*1. Introduction*
• How did you hear about the mobile clinic?
• What do you know about this clinic?
*2. User experiences and needs*
• Why do you use the services of the mobile clinic?
• What led you to use these services?
• Can you talk to me about one of these visits to the clinic?
• What was your need?
• How did you feel during this visit?
• How were your needs met?
• What could have been done differently?
*3. Perceptions*
• What is your perception of access to healthcare services?
• What importance do you attach to a resource such as the mobile clinic?
*4. Recommendations*
• Should the clinic’s health services be improved?
• Do you have any advice for [organisation’s] nurses? Which ones?
• Do you have any recommendations to promote better access to healthcare for people experiencing homelessness?

**Table 2.** Participant characteristics (*n* = 12).

Participants	Gender	Group age in years	Language	Ethnicity
P1	Male	40–55	French	White
P2	Male	65–70	English	White
P3	Male	45–50	English	White
P4	Male	45–50	English	White
P5	Male	65–70	French	White
P6	Male	60–65	English	White
P7	Male	50–55	French	White
P8	Male	45–50	English	White
P9	Male	65–70	French	White
P10	Male	30–35	French	White
P11	Female	50–55	Inuktut/English	Indigenous
P12	Female	40–45	Inuktut/English	Indigenous

In addition to interviews, we drew on non-participant observation. Approximately 70 hours of field observation was conducted by authors IRS, EPG and HEBA concurrently to the interviews. Observation provided an opportunity for immersion in the environment, to become a familiar face, and gave us a better understanding of the ways of doing things and intervening in the field. We used an observation guide (Table 3) developed according to the items proposed by Peretz (2004) and took field notes, as recommended by Emerson et al. (2011). During the observation sessions, we studied interactions outside the mobile clinic, and, on some occasions, we observed interventions inside the clinic during appointments with service users.

**Table 3.** Observation guide.

• Context (location, weather, time of day) • Description of the situation observed (issues, specific culture, situations, behaviours) • Interactions (relationship between actors, language, communication)

### Data analysis

The empirical material was qualitatively analysed according to the steps recommended by Madison (2019). For coding, field notes and transcripts were reread line-by-line to ensure fidelity to the data. Codes were placed in the margins of the text to summarise the central ideas. During the coding process, meetings were held with the co-authors to discuss the choice of codes and the progress of the analysis. A list of codes was drafted and discussed to reach an intercoder agreement (Cascio et al., 2019). In the data categorisation stage, the selected codes were grouped according to their similarity or divergence. Categories and subcategories were juxtaposed to observe similarities and contradictions across the data. At this stage, the empirical material was integrated into concept maps to further schematise the data corpus and arrive at a more abstract interpretation of the data (decontextualisation process). The categories were then compared to the literature on the phenomenon under study.

### Trustworthiness

In this critical ethnography, we respected the scientific criteria of authenticity, reflexivity and positionality proposed by Madison (2019). In terms of *authenticity*, we conducted several hours of observation and various meetings with the actors in the field to have a more detailed understanding of the environment. As for *reflexivity*, we kept a reflexive journal and reread the interview transcripts several times to better immerse ourselves in the participants’ testimony. Throughout the research, we were aware of the influence that our posture as researchers could have in a particular environment such as the street. *Positionality*: The authors have experience as mental health and psychiatric nurses and work in the academic community as scholars. We recognise that this social position may have influenced the process of interpreting the data. Finally, we are aware that we have studied a phenomenon that we have not experienced ourselves.

## Results

Out of the data analysis emerged three main conceptual categories: (1) *worrisome health and social needs*, (2) *non-use of healthcare* and (3) *what connects us to health services*. We will describe each of these categories, presenting both quotes from participants and excerpts from the field notes.

### Category 1 – Worrisome health and social needs

This category focuses on health and social needs reported by participants. The needs relate to physical/mental health and psychosocial issues. For many, these different spheres are interrelated and affect all aspects of their lives. In terms of health, the participants we met highlighted various physical problems, such as infections (e.g. sexually transmitted and blood-borne infections), injuries and chronic illnesses.

The data shows that health problems are often worsened by social disaffiliation, which refers to a severing of ties between individuals and public services. Disaffiliation can take many forms, including isolation, stigmatisation, eviction and inability to work. The process of disaffiliation further compounds their social and health needs.

There are some [other people experiencing homelessness] who are reclusive. They don’t talk to us. They do their own thing. They suffer more in mental health and everything. It’s hard. (P10)

Moreover, in the interviews many reported the stigma they suffered. Stigmatisation is a reality within the healthcare system and, more generally, in society. It is related to social attitudes concerning people who live on the street, their social identification, and their ethnic origin. One participant mentioned her Indigenous origins as a reason for discrimination in the healthcare network.

Maybe they were [the nursing staff in the emergency department] thinking I’m Inuk, and they refused me a little bit. Like, longer waiting for me [at the emergency]. Yes, I think. (P12)

In the context of disaffiliation, the participants talked about the social issues, as well as the physical and mental health problems that affect them. These issues are likely to increase the precariousness and stigmatisation they must deal with, in addition to having a negative impact on their health condition.

[I had] my first AA meeting, cuz my mind is cluttered. I got more problems. I got problems with going to court. All kinds of what I don’t need, right? (P6)

### Category 2 – Non-use of healthcare services

#### Difficulty accessing institutional services

According to the participants, a number of factors make it hard to access institutional services. Various barriers were identified during the interviews, in particular, the requirement of a health insurance card. The fact of not having a valid card prevents many people living on the street from going to an emergency department (ED) or medical clinic. Administrative requirements in hospitals are a barrier to seeking help, as is the fear of having to pay for healthcare (e.g. dental care and prescription drugs). The mobile clinic’s services and their simpler administrative process, with no health card requirement, encourages people to seek help.

I had lost my wallet and everything in it. So I didn’t have a health insurance card. So, it was a good fit. [The clinic] was here. I didn’t have to run. (P1)

The issue of distance from services was also raised as another barrier to access: having to travel by car or public transport discourages many people from going to the hospital. On the other hand, having access to health services close to where they are is a facilitating factor. All the participants described the long wait times in hospitals as a major issue. These delays may cause people to leave the ED or avoid going to the hospital at all.

They take so long, to go, to wait. I don’t like hospitals. (P11)

We don’t always feel like consulting. We are always, sometimes, there are people that . . . there are homeless people who are having a hard time in clinics, and waiting in a waiting room among citizens. They are people who feel judged. Some people don’t always want to go. (P9)

In contrast, wait times for the mobile clinic’s services are much shorter, according to the participants we met. The wait is generally a few minutes, depending on the number of clients and the time of day.

It’s like a ten-minute wait. I got it. That was it. (P6)

At the emergency, they take their time. They go see another patient before you, and this and then. With the mobile clinic, you go right up to them. They reach you. They ask you: ‘What’s wrong?’ And you tell them what’s wrong. And then, they deal with it right there and then. It’s like with the doctors, they take their time, do whatever they have to do before anything else. And then, by the time they get to you, there’s no point in . . . what’s the point of staying? (P8)

Although the wait time is short, some people may still not want to wait the few minutes at the clinic. The observation notes indicate that for these people, they want a direct response to their need when they arrive, without having to wait.

Another patient in his 30s arrived at the same time as the other client. This third client is known to the nurse at the clinic. Since nurses can only provide care to one client at a time, this other client said he would come back a little later, but he didn’t come back until we left. (Field notes)

Another concern, when it comes to barriers, is the lack of continuity and integration between health services (e.g. between mental health and physical health services) which discourages many from seeking help. Participants mentioned that more people would seek assistance if they knew they would receive regular follow-up, close to their living environment, without administrative constraints. Lack of nursing staff and caregiver turnover rate were also mentioned as impediments to access and continuity of care.

That’s the problem with everybody when you’re in the health system, when you’re in the network. Sometimes, even when my caseworker on the treatment team changes, it’s like, well okay. You have to start all over again. With the doctors, it’s the same thing. (P1)

The services of the mobile clinic can, to a certain extent, compensate for this lack of continuity because of their role of referral and orientation throughout the vast and complex healthcare network. Indeed, the role of the nurses and workers at the mobile clinic is to prevent people from ‘falling through the cracks’.

## Putting off going to the hospital

The participants stated that, faced with administrative and attitudinal barriers, people experiencing homelessness tend to put off going for help and treatment. They seek services only when their problems are perceived to be critical, which explains the urgency and last-resort nature of their requests for help. Having lived through a multitude of adverse experiences with institutional services, participants qualified themselves as being in day-to-day survival – rather than prevention and health promotion – mode. Their conception of health and other immediate problems therefore significantly diverges from that of the general public.

It’s very important, because they [the mobile clinic] reach out to people who wouldn’t go to the hospital. If I’m in danger of dying, I might go, if not . . . (P10)

I’m not a guy who goes to the hospital. I’ll go to the hospital really in the extreme emergency. (P9)

We also observed that people are reluctant to go to the hospital, even though their health condition requires it, as illustrated here:

A man came to meet the nurse. He had a bandage on his hand. He was tense and irritable. He would not give his name and date of birth. He was seen by the nurse in the clinic, his bandage was removed, the client had a wound that was bleeding heavily. The nurse advised him to go to the emergency, but he refused, so she cleaned the wound and bandaged it as best she could. The client would not explain how he got hurt. (Field notes)

In this regard, one participant explained why people living on the street do not feel the need to seek health services. They are more likely to ‘endure’ their health problems and illnesses.

The people in the street are not going to go there . . . Why don’t they go? It’s often personal. They don’t want to. Do you understand what I mean? Unless, like I told you, they’re bleeding. Or if they have a physical injury. When it’s a physical injury, when it’s permanent, when it becomes chronic . . . But, apart from that, the homeless people I know, they don’t really use the hospitals. They try to get through their injuries . . . They don’t give a damn about their health. (P7)

### Category 3 – What connects us to health services

In our analysis, we define affiliation mechanisms as the interventions and approaches that are specifically designed to promote relational and social links with a community. This can include formal or informal support, and treatment provided directly in the living environment of people experiencing homelessness. Affiliation mechanisms can help to better support people in their care trajectory.

#### Outreach services

According to the participants we met, the outreach services to which they have access are diversified and operate in an intersectoral perspective (e.g. community health nurses affiliated with hospitals, street workers, social workers, health professionals, etc.). During the interviews, we asked participants about the main aspects of the outreach services specifically provided by the mobile clinic. In contrast to the previously mentioned barriers to access, participants emphasised the importance of the clinic’s broad availability and easy accessibility. As such, the clinic’s presence in the living environment is key to its success. The informal and unrestricted nature of nursing services were also greatly appreciated.

It is without appointment. It happens in the street. It’s live up. (P9)

They come around, help people. (P3)

Reaching out to people where they are is another daily practice in which the mobile clinic goes into the field to provide services directly to people in parks, subways and nearby streets:

Afterwards, there was a period when there were no consultations, so the nurse and the street worker went to do some outreach in the surrounding streets on foot. (Field notes)

The mobile clinic, like other homelessness agencies, is seen as a ‘safe space’, where people can feel less vulnerable and more comfortable to speak openly. During the observation periods and discussions with caregivers we heard women participants in particular mention appreciating the safe space aspect of the clinic. Also, several participants mentioned the clinic’s approach, stating that the caregivers and nurses consider the person as a whole, from a holistic perspective.

The nurse was there, and checked me, if I have anything. It was good. (P12)

Here [at the mobile clinic], it’s click-clack, it’s over. An initial, your date of birth. Then they go according to people’s needs. It meets a lot of needs. I think 100% of the needs are addressed. Whereas in a medical clinic, it’s not 100% of the needs. Yes, you may have more of a professional diagnosis, but . . . (P9)

In addition, active listening was mentioned by some participants as a practice that helps to build trust and makes them feel respected.

They listen to what you have to say: ‘What do you need?’ Then, depending on your answer, they will make recommendations. Then, it’s up to you to take it . . . to take the advice they give you so that you’ll be OK, your health will be better. (P5)

They were nice. They listened. (P11)

In contrast, participants reported that, in institutional settings, when homeless people seek help for health problems they are often not listened to.

No. No. There is no such listening. (P1)

In terms of affiliation mechanisms, our analysis shows that it is important to consider a person as a unique individual. Being treated as a whole person and having one’s immediate needs met fosters greater motivation to be involved in self care and treatment. Participants also emphasised the nurses’ professionalism as an essential value.

They were all real professional, quiet. (P4)

We can see that the bond of trust that develops with regard to the clinic’s services is based on a relationship of respect and collaboration. The development of this bond of trust makes it possible for people to be more inclined to talk about their problem and, eventually, to reconnect with the more conventional healthcare network.

#### Referral role

Participants also mentioned the mobile clinic’s referral and liaison role. It involves informing, guiding, or taking people to the appropriate health service, whether it is the ED, a walk-in clinic or a pharmacy. In some cases, this can help to prevent more serious complications in the condition of people who, as we have seen, are reluctant to consult for their health needs. Through this referral role, caregivers try to develop or maintain people’s affiliation links with institutional services.

They give us good referrals. So I use them for this. I know they’re not the ones who are going to treat all my problems. But they’re definitely going to refer me. (P7)

#### Support

Support (whether professional or peer) emerges as a key concept in all the collected data. For some people, having access to a social worker or caseworker facilitates psychosocial and administrative procedures, such as applying for a health insurance card.

[The street worker] would come and check on me at my house to see if I felt down . . . He knew I wasn’t eating . . . [He] was fantastic. He did everything. He did all the important things with me. (P6)

Another participant reported the importance to her of the advocacy and support her practitioner provides when she visits the clinic or health services, to help her ask questions and feel more at ease:

Sometimes, when I’m scared, I bring my caseworker. (P11)

Being involved with a treatment team in the community or clinic is also considered by many to be a strong bond of affiliation, ensuring regular follow-up on their health status.

This is the ACT team. It stands for Active Community Treatment. It’s a weekly, or regular, follow-up. It was with their help that I got an apartment yesterday. Yesterday, I moved into a really nice studio. And, it’s . . . they help me, because I help myself. I take my injections. Then, I’m more stable. With that, I can go further, with them. (P1)

Participants also mentioned social support (i.e. the presence of people round you) in addition to formal support from health professionals, as being important to their mental health and well-being.

In emergencies, you need help. To be helped, to be guided and supported to get better. (P5)

During the field sessions, we observed that people come to the clinic with their peers, with friends, or as a couple. One finding was that people helped each other: some recommended the clinic to their peers, they accompanied them and stayed with them while they waited for an appointment. The clinic’s services are publicised by word of mouth among the people on the street. In addition, participants stated that seeing peers getting treatment and feeling better overall has a ripple effect: it may also encourage them to seek help and counselling.

Word got out quickly among the people that we were there. (Field notes)

## Discussion

Our analysis shows that participants access the mobile clinic not only because of their health problems, but also due to their overall situation of precariousness (e.g. loss of housing, malnutrition, difficulty accessing employment and uninformed about available assistance programmes). They therefore present multiple problems, in terms of physical and mental health as well as psychosocial well-being. Lack of work, a precarious living environment and a weak support network have direct impacts on people’s health (Luchenski et al., 2018).

Indeed, for these participants, we see that substance use, stress and exacerbation of health problems are interrelated. In this, our results concur with the findings of Campbell et al. (2015) in relation to the significance of social determinants of health among people experiencing homelessness (e.g. social protection and assistance, employment, food security, social exclusion and access to housing and affordable health services).

Many of those who visit the mobile clinic are in a situation we would characterise as social disaffiliation and are reluctant to use more conventional health services. The collected data reveals obstacles including previous negative experiences, stigmatisation in the healthcare system and numerous access barriers (waiting times in ED, distance, lack of a valid health insurance card, administrative burden and attitudinal barriers of care providers). These barriers create inequities in access to health services, which may compound people’s health and social problems. The study of Bhui et al. (2006) as well as that of Daiski (2005), conducted with users of a mobile health bus in the Toronto area, also raise the reality of stigma and prejudicial attitudes in care settings. Similarly, several other studies have documented these barriers to accessing services for people experiencing homelessness (Campbell et al., 2015; Hudson et al., 2010).

Our analysis also reveals that non-use of health services is pervasive on the street. Non-use can be explained by the lifestyle imposed by the phenomenon of homelessness. People will seek out the services of the mobile clinic when the situation becomes extremely serious, as a last resort. Outreach nursing practices, such as those of the mobile clinic, are part of a community-based approach, where services are offered directly in the living environment, with the fewest possible constraints. We may therefore conclude that such health approaches are appreciated and used primarily because they enable the delivery of care ‘right now’, in response to the immediate needs of people who are otherwise reluctant to concern themselves with their health.

In this regard, Warin (2018, 2019) states that people in vulnerable situations face so many barriers that they prefer not to seek help. This author refers to the concept of ‘non-take-up’, defined as when vulnerable populations drop out of systems that ensure the protection of social rights (e.g. health, employment, social assistance, social services) to which they are entitled. Non-take-up can be explained by social isolation, stigma and the disaffiliation process. In terms of support, intentional and responsive accompaniment is necessary to prevent non-use of healthcare (Warin, 2019). As our study shows, accompaniment can take the form of either informal or formal support. Warin’s ideas concord with the writings of critical sociologist Robert Castel, who focuses on the support network and social programmes required for people experiencing homelessness. Castel (2000) affirms that expanded and adequately subsidised state support is needed for populations trapped in the ‘zone of disaffiliation’.

Our results align with other studies on nurse-led clinics (Randall et al., 2017; Savage et al., 2008). The literature we consulted shows that they result in increased access to healthcare services, a largely positive level of satisfaction and positive outcomes for service users (reduced risk of health complications, a feeling of inclusion and of being listened to and greater self-awareness of one’s health). In these clinics, human contact and listening are important aspects raised by participants (Daiski, 2005). The respectful approach of openness, non-judgment and consideration is also mentioned in the literature (Warren et al., 2021).

We found that the referral role the mobile clinic plays in partnership with street workers and community organisations enables reaffiliation with the healthcare network and institutional services (e.g. hospitals, ED and medical clinics). The liaison role enables a more fluid and rapid access to healthcare, in addition to reducing the apprehension and mistrust that people have towards institutional services. A similar finding emerges among the studies we consulted. Outreach nursing services are considered an important referral, helping to reduce the need for hospitalisation (Daiski, 2005; Roche et al., 2017).

By adhering to a critical methodological approach, this research goes beyond the purely clinical aspects of nursing interventions in homelessness while also addressing the social and political dimensions associated with such interventions. The relevance of this research is described by the urgency to better understand how nursing outreach work is received and experienced by people experiencing homelessness and how such work contributes to the quality of care and services offered in the community. Our findings are therefore critical to influence policymakers on strategies for the care and services of these vulnerable individuals. The outcomes of this study can also be used to raise awareness among nursing students about the issues of care for marginalised people.

### Limitations

In this study, the limited number of female participants compared to males can be seen as a limitation in the recruitment process; it would have been relevant to obtain testimony from more people with different gender identities. The same is true for the low diversity in terms of ethnicity. Finally, we are aware that our fieldwork was conducted in a specific context, that of a large Canadian city where healthcare is public and universally covered for the population.

## Conclusion

People experiencing homelessness live in very precarious conditions that cause and aggravate health problems requiring accessible care. Nurse-led clinics, which offer outreach services, are an essential method for ensuring access to healthcare for people experiencing homelessness. Our study provides a better understanding of how the work of nurses who accompany them contributes to reducing the consequences of disaffiliation from the traditional health system. These services are very much appreciated, but there may be a risk that they will contribute to keeping homeless persons outside the traditional system, leading to a form of ghettoisation. Further research is needed to decide whether outreach services should continue to grow or whether action should instead be taken to improve access to the usual services, to ensure that homeless persons have access to the services they need.

**Key points for policy, practice and/or research**

- Different strategies have been developed to address the health needs of people experiencing homelessness, such as nurse-led clinics and mobile health clinics.
- The social disaffiliation of people experiencing homelessness is also embodied in their relationship to health services.
- Nurse-led clinics meet needs that go far beyond health issues.
- Homeless people preferred more nurse-led clinics rather than better access to usual services.
